# Circulating MicroRNA-499 as a Diagnostic Biomarker for Acute Myocardial Infarction: A Meta-analysis

**DOI:** 10.1155/2019/6121696

**Published:** 2019-05-02

**Authors:** Jingyi Zhao, Hairong Yu, Peng Yan, Xiaohui Zhou, Ying Wang, Yinhui Yao

**Affiliations:** ^1^Department of Functional Center, Chengde Medical College, Chengde 067000, China; ^2^School of Basic Medicine, Chengde Medical College, Chengde 067000, China; ^3^Department of Pharmacy, Affiliated Hospital of Chengde Medical College, Chengde 067000, China

## Abstract

**Background:**

Recent studies have shown that circulating microRNA-499 could be a powerful biomarker of acute myocardial infarction (AMI). Interest in circulating microRNA-499 for detecting AMI is increasing rapidly. To evaluate the diagnosis of circulating microRNA-499 for AMI, this study was performed.

**Methods:**

We searched PubMed, Embase, and the Cochrane Library for studies published up to December 31, 2018, as well as the reference lists of relevant studies. Studies were included if they assessed the accuracy of blood circulating microRNA-499 or cardiac troponin T (cTnT) for AMI and provided sufficient data to construct a 2 × 2 contingency table. Extracted data were analysed for sensitivity, specificity, diagnostic odds ratio (DOR), and summary receiver operator curve (SROC) analyses. Prespecified subgroup analysis and metaregression were also performed.

**Results:**

Fourteen studies including 3816 participants were included in this meta-analysis. The overall pooled sensitivity and specificity were 0.84 (95% CI: 0.64-0.94) and 0.97 (95% CI: 0.90-0.99), respectively. The area under the SROC curve (AUC) was 0.98 (95% CI: 0.96-0.99). The studies had substantial heterogeneity (*I*^2^ = 98.74%). Seven studies also used cTnT as a marker for the diagnosis of AMI. The overall pooled sensitivity and specificity of cTnT were 0.95 (95% CI: 0.87-0.98) and 0.96 (95% CI: 0.85-0.99), respectively. The area under the SROC curve (AUC) was 0.99 (95% CI: 0.97-0.99). The DOR of circulating miR-499 and cTnT were 188 (95% CI: 19-1815) and 420 (95% CI: 86-2038), respectively. Metaregression analysis suggested that specimen and healthy controls were the main sources of heterogeneity. No publication bias was suggested by Deeks' regression test of asymmetrical funnel plot (*t* = 0.85; *p* value = 0.41).

**Conclusion:**

The results showed that circulating microRNA-499 is a reliable biomarker for diagnosing AMI patients.

## 1. Introduction

Acute myocardial infarction (AMI), which is the most common cause of death worldwide, is an acute necrosis caused by continued severe ischemia of the myocardial tissue. In 2020, an estimated 16 million people will suffer from AMI, and about 23 million people will suffer in 2030 [1]. Thus, rapid and accurate diagnosis of AMI plays a crucial role in therapy and prognosis, which would reduce morbidity and mortality of this disease. Currently, the usefulness of myocardial circulating biomarkers, such as cardiac troponin T (cTnT) and creatine kinase MB (CK-MB), maximizes the benefits of revascularization therapy, as the most effective biomarkers in clinical practice [2, 3]. However, there is still a relatively low diagnostic accuracy earlier than 4-8 h after the onset of AMI. The biomarkers of cTnT and CK-MB are likely to increase, whether AMI occurs or not [4, 5]. Previous studies also showed that the significant levels of cTnT were identified only around 6 hours, resulting in new biomarkers for extremely early diagnosis [6]. Thus, exploring novel biomarkers with both high sensitivity and specificity for AMI is urgently required.

MicroRNAs (miRNAs/miRs), a class of small (19-25 nucleotides) noncoding RNAs, are important posttranscriptional regulators of numerous biological processes including cell growth, proliferation, differentiation, and apoptosis [7]. The expression profile of miRNAs was found in tissue-specific or cell-specific distributions [8]. Recently, a number of studies reported that microRNAs are circulating in plasma/serum and can be considered as biomarkers in cardiovascular disease [9]. MicroRNA-499, a member of the microRNA family, has been shown to be expressed in myocardium and skeletal muscle in mammals [10]. Previous to this, some studies have showed that the sample of blood from AMI patients with the high expression level of microRNA-499 can be detected earlier [11]. So, its correlation suggests that the interplay of the specific circulating microRNA-499 and the development of cardiovascular disease might be useful diagnosis biomarkers and therapeutic targets for AMI [12]. Owing to studies with small sample sizes and controversial issues, we conducted the current evidence regarding the use of circulating microRNA-499 for the identification of AMI by performing a meta-analysis.

## 2. Methods

### 2.1. Search Strategy

Give attention to the three electronic databases (PubMed, Embase, and Cochrane Library) that clinical studies reported on the diagnostic accuracy of circulating microRNA-499 for AMI and published through December 31, 2018. The search keywords were “circulating microRNA-499” in combination with “acute myocardial infarction” or “AMI”. There were no publication dates or language restrictions. The reference lists of all relevant review articles also were retrieved, and only raw data were used for further analysis. All relevant articles are based on previously published studies; thus, unpublished studies were not searched.

Studies were considered eligible if they were case-control studies that reported the diagnostic accuracy measures of the circulating microRNA-499 (or cTnT) in patients with AMI as the case group and non-AMI patients as the control group. Eligible studies contained sufficient information for the construction of 2 × 2 contingency tables to assess the diagnosis value of circulating microRNA-499 in AMI patients in the meta-analysis. The excluded studies are as follows: (a) review articles, (b) case reports, (c) editorials, (d) conference abstracts, and (e) clinical protocol.

### 2.2. Quality Assessment

We systematically assessed the quality of the studies included in the diagnostic meta-analysis with the Quality Assessment of Diagnostic Accuracy Studies-2 (QUADAS-2) tool [13].

### 2.3. Data Extraction

According to a standardized form, two reviewers independently extracted data from the eligible studies and recorded information. If there was a disagreement regarding a particular article's eligibility for the analysis, it was better to be resolved by consensus. The elements of extracting data from the included studies are the author's name, the year of publication, the country of origin, the number of cases and controls, biomarkers, and the sensitivity and specificity of the indicated biomarker for the diagnosis of AMI.

### 2.4. Statistical Methods

The MIDAS module of STATA 14.0 (StataCorp, College Station, Texas, USA) and Meta-DiSc 1.4 (XI Cochrane Colloquium, Barcelona, Spain) were used for statistical analysis. In order to assess the overall diagnostic value of miR-499 in distinguishing AMI patients from controls, the pooled sensitivity, specificity, positive likelihood ratio (PLR), negative likelihood ratio (NLR), diagnostic odds ratio (DOR), the bivariate summary receiver operator characteristic (SROC) curve, and the area under the curve (AUC) were calculated. Statistical heterogeneity among the studies was assessed using *I*^2^ statistics. Values of 25%, 50%, and 75% for the *I*^2^ test were considered low, medium, and high statistical heterogeneities, respectively. Metaregression analysis was performed to find the effect of potential heterogeneity in sensitivity and specificity. To assess the publication bias of the included studies, we performed Deeks' regression test of funnel plot asymmetry [14]. A *p* value of <0.05 was considered statistically significant.

## 3. Results

### 3.1. Data Selection and Study Characteristics

**Figure 1:**
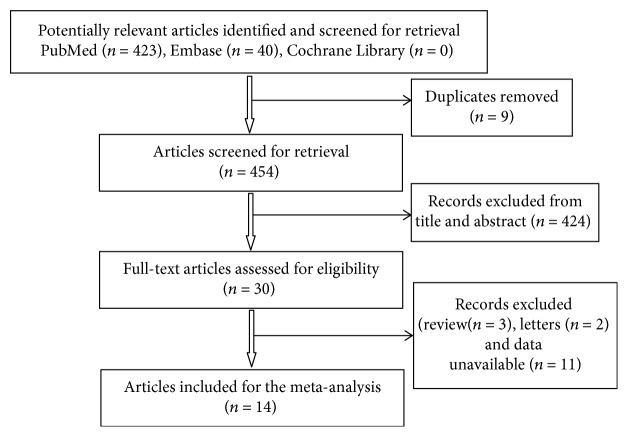
Flow chart of systematic literature search.

Our initial search yielded 463 articles, of which 424 were eliminated after screening the title and abstract. We scrutinized 30 studies for full-text review and identified 14 studies that fulfilled our eligibility criteria (Figure 1). Overall, a total of 3816 patients were included from the 14 studies, 1989 of whom had AMI and 1732 of whom had non-AMI [15–28]. Among these 14 studies, 11 used plasma samples, whereas the rest used serum. The included studies were performed in China, Luxembourg, Sweden, Egypt, and Greece.  Table 1 presents the detailed characteristics of each subject.

### 3.2. Quality of the Included Studies

**Figure 2:**
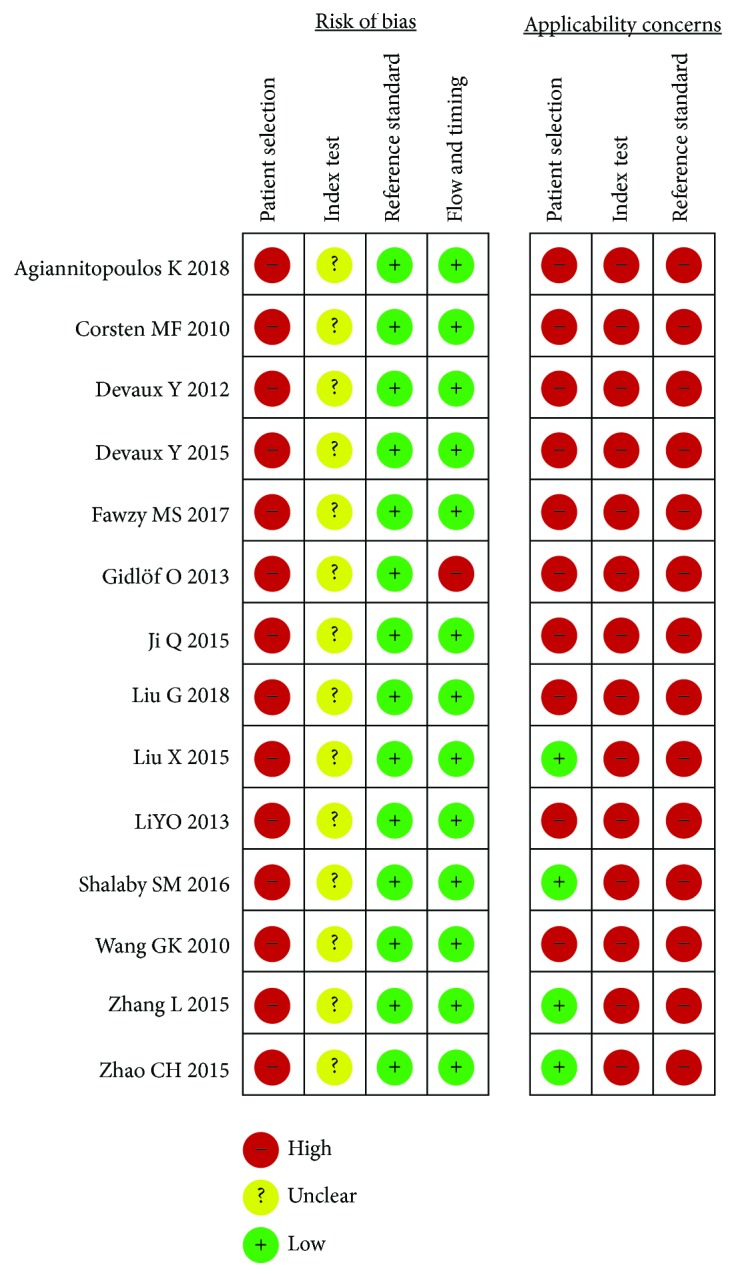
Risk of bias and applicability concern graph.

QUADAS-2 quality assessment of the included studies and the results of critical appraisal are shown in Figure 2. The quality of all studies was considered mediocre. All the included studies did not describe fully the methods of patient selection, most notably with respect to whether a consecutive or random sample of patients was enrolled. We found no mention of a threshold of circulating miR-499 for AMI patient in terms of its prespecificity in all the included studies. Many studies did explicitly state severity of the target condition, demographic features, and the presence of differential diagnosis [15–21, 26–28].

### 3.3. Diagnostic Accuracy

**Figure 3:**
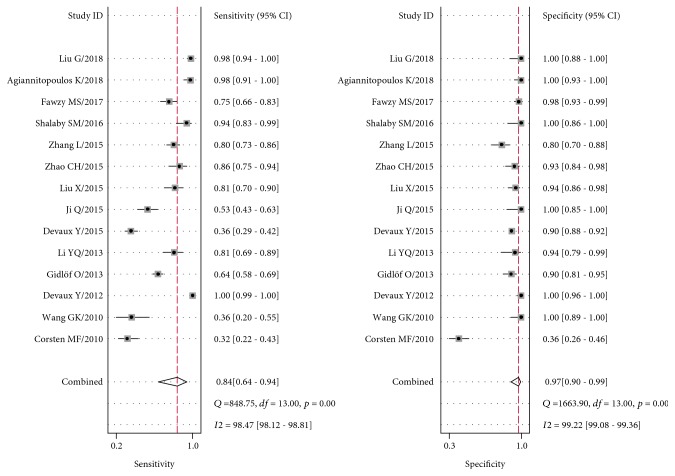
Forest plots for sensitivity and specificity for studies using circulating microRNA-499 to detect among patients with acute myocardial infarction.

**Figure 4:**
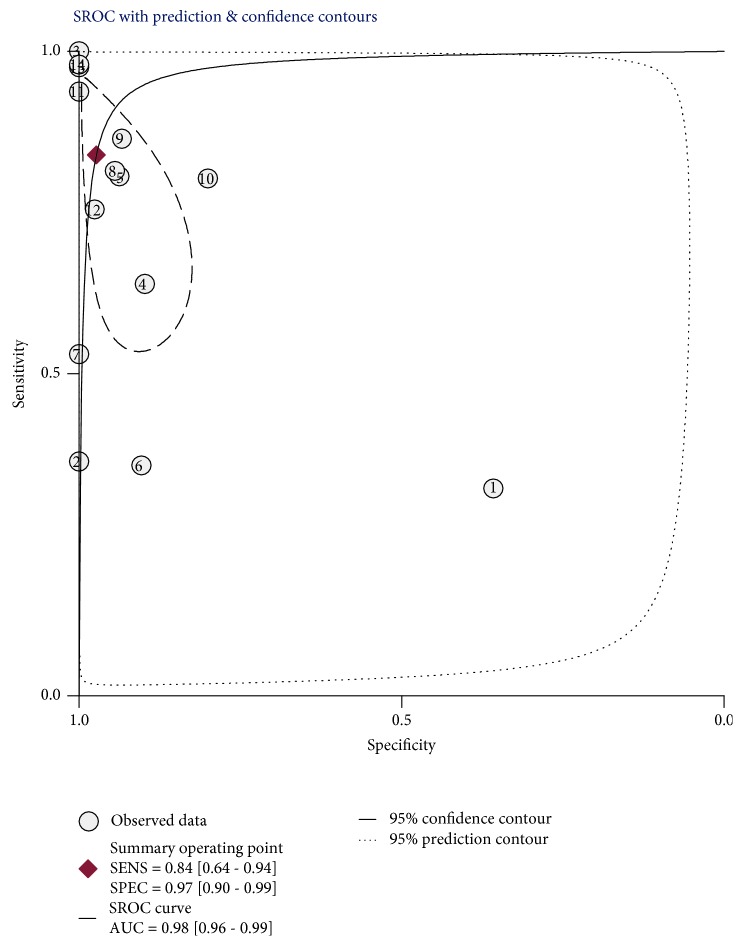
Symmetrical summary receiver operator curve (SROC) of circulating miR-499 for all 14 studies.

The pooled sensitivity and specificity estimates for the circulating miR-499 were 0.84 (95% CI: 0.64-0.94) and 0.97 (95% CI: 0.90-0.99), respectively (Figure 3). The pooled PLR was 31.1 (95% CI: 6.9-140.3), and the pooled NLR was 0.17 (95% CI: 0.07-0.41). The DOR was 188 (95% CI: 19-1815), indicating better discriminatory test performance. The area under the SROC curve for circulating miR-499 was 0.98 (95% CI: 0.96-0.99), indicating a high accuracy (Figure 4). The pooled sensitivity of cTnT for the diagnosis of AMI was 0.95 (95% CI: 0.87–0.98), and the pooled specificity of AMI for the diagnosis of AMI was 0.96 (95% CI: 0.85–0.99). The pooled PLR was 23.5 (95% CI: 5.8-94.8), and the pooled NLR was 0.06 (95% CI: 0.02-0.14). The area under the SROC curve was 0.99 (95% CI: 0.97-0.99), and the DOR was 420 (95% CI: 86-2038). Taking all of the findings into consideration, circulating miR-499 can be provided with highly diagnostic accuracy as well as cTnT to distinguish AMI from non-AMI.

### 3.4. Heterogeneity Analysis and Subgroup Analysis

The results of significant heterogeneity were observed among included studies. For all 14 studies, the heterogeneity (*I*^2^) was 98.47% (sensitivity) and 99.22% (specificity).

**Figure 5:**
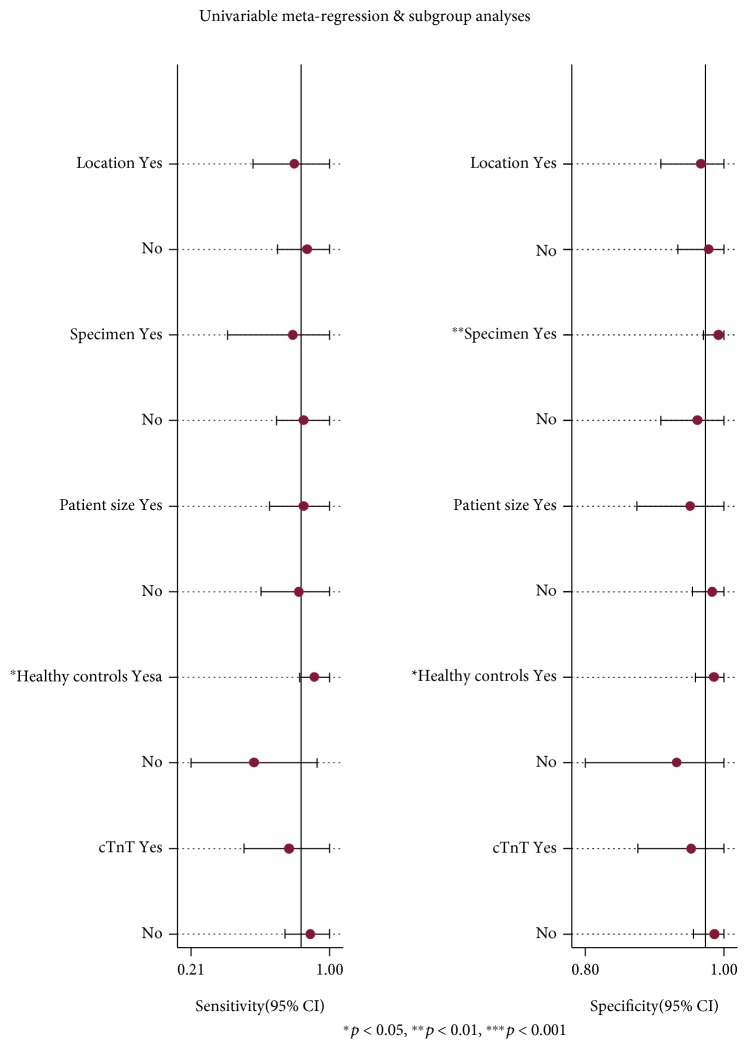
Univariable metaregression and subgroup analyses.

The source of heterogeneity was completely examined by metaregression analysis using study covariates such as location, specimen, patient size, healthy controls, and cTnT. To examine the source of heterogeneity completely by making each covariate associate with logit (sensitivity) and logit (specificity), metaregression analysis showed that healthy controls were the most important of sources of heterogeneity (Figure 5). When exploring the source of heterogeneity by making study covariate associate with logit (specificity), metaregression analysis showed that the specimen significantly accounted for the heterogeneity for specificity. According to the specified groups, a subgroup analysis was performed to assess differences in diagnostic accuracy by healthy controls. The nine studies that reviewed healthy controls showed a high-pooled sensitivity (0.91, 95% CI: 0.83-1.00) and specificity (0.99, 95% CI: 0.96-1.00). In contrast, the rest of the five studies performed in nonhealthy controls showed a low-pooled sensitivity (0.57, 95% CI: 0.21-0.93), but the specificity was high (0.91, 95% CI: 0.80-1.00). Subgroup analysis by plasma showed a low-pooled sensitivity (0.85, 95% CI: 0.70-1.00) but a high-pooled specificity (0.96, 95% CI: 0.91-1.00). The remaining 3 studies of serum of the pool had sensitivity and specificity of 0.79 (95% CI: 0.42-1.00) and 0.99 (95% CI: 0.97-1.00), respectively.

### 3.5. Publication Bias

**Figure 6:**
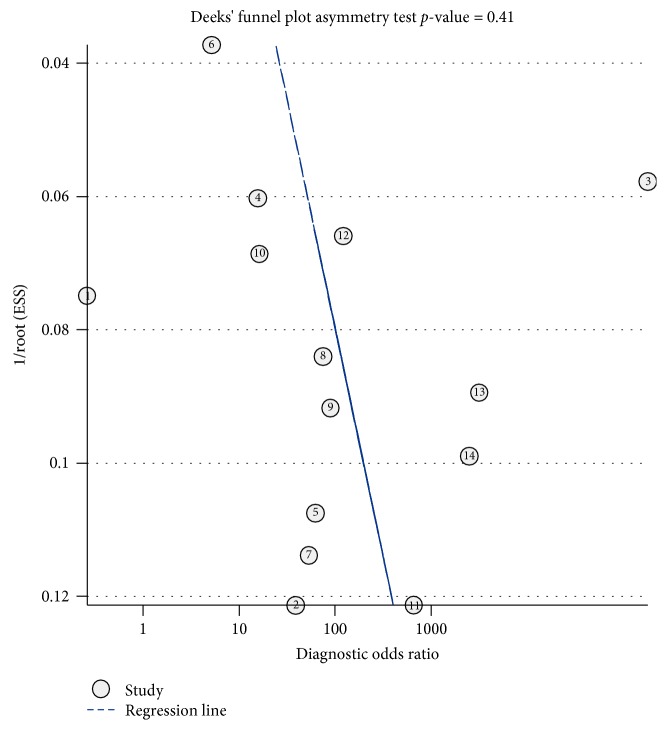
Deeks' funnel plot.

Deeks' funnel plot asymmetry test suggested no potential publication bias with asymmetry in our study data (*t* = 0.85; *p* value = 0.41) (Figure 6).

## 4. Discussion

Circulating miR-499 can differentiate effectively between AMI and non-AMI. Previously, two meta-analyses have revealed that the expression level of circulating miR-499 was a better biomarker for identifying patients with AMI [22, 29].

In a meta-analysis from 2015, including 8 studies published between April, 2010, and October, 2015, Liu et al. confirmed that the predictive values of plasma miR-499 for AMI were better than those of the miR-1 and miR-208 [22]. Owing to a relatively small study population being included in this meta-analysis, the heterogeneity of patients from the sample type had no evaluation. Furthermore, a previous meta-analysis indicated that miR-499 had better diagnostic accuracy over other miRNAs (miR-1 and miR-133) [29]. However, the investigators restricted the population to Asian. Therefore, no conclusion can be considered for AMI patients from the different regions.

**Figure 7:**
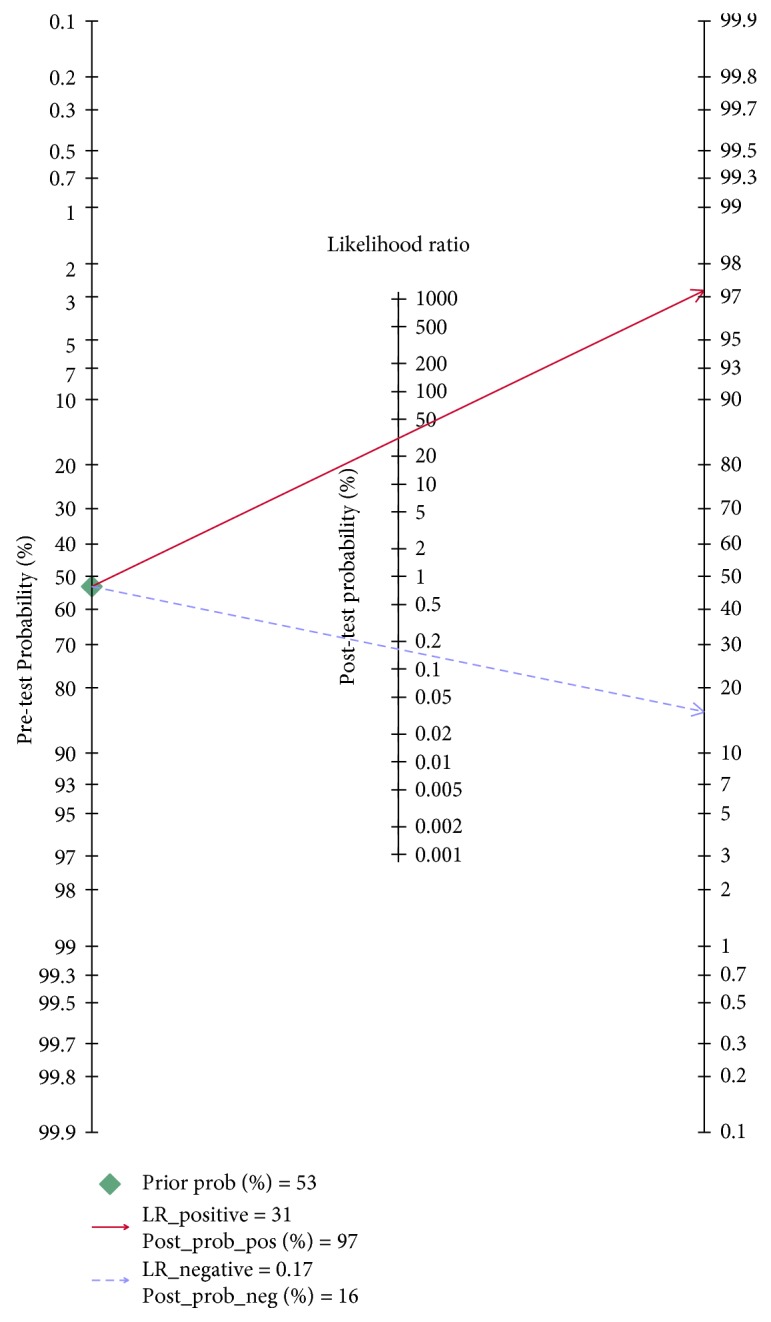
Fagan nomogram of circulating microRNA-499 for diagnosis of acute myocardial infarction.

The results of the current study prove that circulating miR-499 has good sensitivity and specificity for differentiating AMI from non-AMI (0.84 and 0.97, respectively). These results of sensitivity and specificity are similar or even better than those reported in two previous studies [22, 29]. In addition, our results of DOR and AUC for diagnosis of AMI were 188 and 0.98, respectively; these values are higher than the findings of Liu et al. [22] and Wang et al. [29]. In order to be more clinically informative in our results, the pooled LRs were used to estimate posttest probabilities. A PLR of 31.1 implies that a person with AMI has about 31 times more likely to be miR-499-positive than a non-AMI person. The NLR of 0.17 suggested that a person with AMI is 17% if the circulating miR-499 is negative. When the pretest probability of AMI was 53%, the pooled PLR [30] increased the posttest probability (positive predictive value) to 97%. Likewise, the pooled PLR (0.17) reduces the posttest probability (negative predictive value) to 16%. Therefore, only 29 out of 30 miR-499-positive patients had the chance to be diagnosed as AMI. In addition, only 1 out of 6 miR-499-negative patients may eventually have AMI (Figure 7).

DOR is a single index of diagnostic test performance, which has nothing to do with the disease prevalence rate. The pooled DOR of miR-499 included studies that were lower than cTnT (188 vs. 420). The differences found in those included studies may be caused by several reasons. First, because of the small number of studies, the results of cTnT tend to overestimate the effect size. Another possible reason was timing of measurement. Recent studies indicated that miRNAs were detected at an earlier stage of AMI and steadily present in circulation, but the cTnT level was very difficult to observe and was below the cut-off value [18, 24]. Therefore, measurements of the cTnT levels were performed at 4-8 h later or as long as clinically indicated, which could increase the diagnostic efficiency of AMI.

There was substantial heterogeneity for miR-499 (*I*^2^ = 97.95%, 95% CI: 95-99) in this meta-analysis. Thus, the source of heterogeneity could be found by several methods, such as threshold effect, publication bias, and metaregression. The result of Deeks' funnel plot asymmetry test showed that no publication bias exists in the analysis, which is not a source of heterogeneity. However, the Spearman correlation analysis (correlation coefficient = -0.6, *p* = 0.023) showed that there was a threshold effect. Although the proportion of heterogeneity was 65% due to a threshold effect, we were unable to discuss this threshold in metaregression because the thresholds of circulating miR-499 were not consistent between AMI and healthy controls. The results of metaregression have shown that the specimen and healthy controls demonstrate the source of heterogeneity from various covariates. Among 5 covariates, we investigated that the circulating miR-499 in plasma have higher sensitivity than that in serum. The reason may attribute it to a coagulation process that affected the expression level of circulating miR-499. Additionally, circulating miR-499 in nonhealthy controls showed that sensitivity and specificity were better than in patients who came from healthy controls. The reason for it may be that circulating miR-499 was nearly undetectable in healthy controls [17, 24], but it increased in MI patients and geriatric non-ST elevation MI patients [18, 31].

In this meta-analysis, limitations should be noted. First, the study characteristics were the differences between research groups (e.g., age, sex, sample collection time, specimen, test method, and location), which may account for the majority of this heterogeneity. However, a relatively small study population limited our ability to detect potential sources of heterogeneity by metaregression. Second, there was s wide variation in sensitivity and specificity which a wide range of cut-offs in the reported circulating miR-499 tests separated patients who had AMI from those who did not. To obtain the most favourable results for diagnostic accuracy, some studies had different thresholds, even when the circulating miR-499 expression level was detected by using the same method. Third, although publication bias was not detected in our analysis, the number of included studies was small. Studies have good results that are more likely to be published, so the diagnostic accuracy of results is overestimated. Despite above limitations, this meta-analysis demonstrates a comprehensive assessment and robust evidence of the diagnostic accuracy of circulating miR-499 for assessing AMI.

In summary, this meta-analysis suggests that circulating miR-499 is of value to AMI. It may be considered for early diagnosis of AMI in emergency. Moreover, these findings indicate that incorporating circulating miR-499 into clinical decision-making has the potential to guide treatment more accurately. Therefore, further studies to formulate a standardized diagnostic criterion and to identify the optimal cut-off values are necessarily required.

## Figures and Tables

**Table 1:** Information of the included studies.

Author	Year	Country	Specimen	Case/control	Biomarkers	Sensitivity	Specificity
Corsten et al. [15]	2010	Luxembourg	Plasma	32/36	miR-499	84	95
Wang et al. [16]	2010	China	Plasma	33/33	miR-499	36.4	100
Devaux et al. [17]	2012	Luxembourg	Plasma	510/87	miR-499	100	100
Gidlöf et al. [18]	2013	Sweden	Plasma	319/88	miR-499	64	90
					cTnT	95	85
Li et al. [19]	2013	China	Plasma	67/32	miR-499	80	94
					cTnT	96	100
Devaux et al. [20]	2015	Luxembourg	Plasma	224/931	miR-499	35.7	90.3
					cTnT	70	96
Ji et al. [21]	2015	China	Serum	98/23	miR-499	53	100
					cTnT	94	82
Liu et al. [22]	2015	China	Plasma	70/72	miR-499	82.1	94
Zhao et al. [23]	2015	China	Plasma	59/60	miR-499	86.37	93.47
					cTnT	93.12	100
Zhang et al. [24]	2015	China	Plasma	142/85	miR-499	80	80.28
					cTnT	100	82
Shalaby et al. [25]	2016	Egypt	Serum	48/25	miR-499	93.4	100
Agiannitopoulos et al. [26]	2017	Egypt	Serum	110/121	miR-499	75	97.2
Fawzy et al. [27]	2018	Greece	Plasma	80/50	miR-499	98	100
					cTnT	94	100
Liu et al. [28]	2018	China	Plasma	145/30	miR-499	98	100

## Data Availability

The data of Table 1 used to support the findings of this study are included within the article (see References).
